# Combined Direct Current Cardioversion and Left Atrial Appendage Occlusion in Patients With Contraindication to Anticoagulation: A Single-Center Feasibility Study

**DOI:** 10.1016/j.jscai.2026.105393

**Published:** 2026-06-02

**Authors:** Lawrence Chen, Tai Pham, Edris Aman, Anthony Khong, Irsa Munir, Tiffany Dong, Muhammad B. Munir, Thomas W.R. Smith, Jason H. Rogers, Gagan Singh

**Affiliations:** Division of Cardiovascular Medicine, University of California Davis Medical Center, Sacramento, California

**Keywords:** antithrombotic therapy, atrial fibrillation, cardioversion, left atrial appendage occlusion, stroke prevention, structural heart interventions

## Abstract

**Background:**

Direct current cardioversion (DCCV) for atrial fibrillation is typically followed by 3 to 4 weeks of oral anticoagulation (OAC) because of atrial stunning and thromboembolic risk, which some patients cannot tolerate. Left atrial appendage occlusion (LAAO) provides an OAC-sparing alternative, but the feasibility of performing DCCV immediately before LAAO has not been well defined.

**Methods:**

We conducted a retrospective single-center study of consecutive patients with atrial fibrillation with relative or absolute contraindications to post-cardioversion OAC who underwent planned DCCV immediately followed by percutaneous LAAO using Watchman FLX or FLX Pro devices. Procedural characteristics, in-hospital outcomes, follow-up imaging findings, and clinical outcomes at 45 days, 3 months, and 1 year were assessed using cardiac computed tomography or transesophageal echocardiography.

**Results:**

Twelve patients underwent concomitant DCCV + LAAO (mean age 75 ± 9 years; 33% women). Bleeding history was present in 67%, with mean CHA_2_DS_2_-VASc and HAS-BLED scores of 3.7 ± 1.1 and 2.1 ± 0.7, respectively. Acute DCCV success was achieved in 11 patients (92%), and LAAO technical success was 100%. Mean procedure time was 48 ± 15 minutes. Follow-up imaging was completed in 12 patients and identified 1 low-risk device-related thrombus (9%), peri-device leak >3 mm in 18%, and no leaks >5 mm. At 45 days and 3 months, there were no deaths, strokes, systemic embolic events, or major bleeding events. At 1 year (n = 7), 1 cardiovascular death occurred without thromboembolic or major bleeding complications.

**Conclusions:**

In this pilot series, a combined DCCV + LAAO strategy was feasible and not associated with excess thromboembolic or bleeding events. Early and intermediate outcomes were comparable to contemporary standalone LAAO experience. Larger prospective studies are needed to further evaluate this OAC-sparing approach.

## Introduction

Atrial fibrillation (AF) is a leading cause of cardioembolic stroke, with the left atrial appendage (LAA) serving as the predominant site of thrombus formation.^1^ Oral anticoagulation (OAC) remains the standard therapy for stroke prevention, and US and European guidelines recommend uninterrupted OAC for at least 3 to 4 weeks following direct current cardioversion (DCCV) because of concerns for atrial stunning and post-cardioversion thromboembolism.^2^^,^^3^ However, many patients who would otherwise benefit from rhythm restoration have relative or absolute contraindications to anticoagulation, including prior major bleeding, frailty, fall risk, or high HAS-BLED scores. In such patients, guideline-directed DCCV is often deferred because it mandates a period of systemic OAC exposure.

Percutaneous LAA occlusion (LAAO) has emerged as an established alternative to long-term anticoagulation for stroke prevention in appropriately selected patients with nonvalvular AF. Randomized trials and large registries have demonstrated that LAAO is noninferior to warfarin for preventing stroke and systemic embolism and may reduce long-term bleeding and mortality.^4^, ^5^, ^6^, ^7^ Contemporary guidelines endorse LAAO in AF patients who are poor candidates for long-term systemic OAC.^2^

The combination of DCCV + LAAO with systematic cardiac computed tomography (CT) follow-up to evaluate device-related thrombus (DRT) or peri-device leak (PDL)—key safety end points that may be more sensitively detected on CT than transesophageal echocardiography (TEE)^8^, ^9^, ^10^, ^11^—has not previously been reported. The present retrospective single-center pilot study evaluates the safety, feasibility, and intermediate outcomes of a combined procedural strategy in which DCCV is performed immediately before percutaneous LAAO in patients with AF and elevated bleeding risk seeking a nonpharmacologic alternative to long-term OAC. This investigation incorporates structured CT-based follow-up imaging to characterize DRT and PDL in this population.

## Methods

### Study design and patient selection

We conducted a retrospective, single-center observational study evaluating the feasibility of a combined strategy of DCCV followed immediately by LAAO in patients with AF and a relative or absolute contraindication to systemic OAC. This study was conducted in accordance with the Declaration of Helsinki and approved by the Institutional Review Board of the University of California Davis Health with a waiver of informed consent because of its retrospective design. The combined DCCV and LAAO approach represents an off-label strategy and was performed under shared decision making with appropriate documentation in the medical record and procedural consent. Consecutive patients undergoing planned concomitant DCCV + LAAO at the University of California Davis Health between November 2023 and October 2025 were included. Patients were eligible if they had (1) documented AF requiring restoration of sinus rhythm, (2) prohibitive bleeding risk precluding post-cardioversion OAC, and (3) suitable LAA anatomy for device-based occlusion. Patients undergoing LAAO without cardioversion or those with incomplete procedural or follow-up data were excluded.

The definition of ”contraindication to oral anticoagulation“ reflects a combination of prior bleeding history, perceived elevated bleeding risk, and patient- and provider-level shared decision making, consistent with real-world LAAO practice. This includes patients with clinically relevant nonmajor bleeding, high fall risk, frailty, or other factors in which long-term anticoagulation is deemed undesirable.

The need for DCCV was determined by a shared decision-making process between the referring cardiologist, implanting physician, and the patient. DCCV was performed in patients with symptomatic paroxysmal or persistent AF and/or suboptimal rate control despite medical therapy. In several cases, rhythm restoration was pursued to improve functional status and quality of life. Combining LAAO with DCCV in the same process was clinically motivated by the desire to eliminate the 3- to 4-week period of mandatory systemic anticoagulation traditionally required after cardioversion because of the theoretical risk of LAA thrombus formation. Instead, patients underwent LAAO during the same procedural episode, enabling avoidance of systemic OAC while still providing stroke prophylaxis and expediting the attempt at restoration of sinus rhythm.

### Procedural protocol

All cases were performed in a cardiac or electrophysiology lab under the direction of a multidisciplinary team. General anesthesia was used in most patients, with TEE serving as the primary imaging modality. A minority of procedures used intracardiac echocardiography as adjunctive guidance. Vascular access, transseptal puncture, and LAA assessment were performed following standard institutional protocols.

### Cardioversion strategy

DCCV timing (pre- or intraprocedural) was left to the discretion of the operator. Energy selection, number of shocks, and need for antiarrhythmic drug augmentation followed routine AF management practice. Acute cardioversion success was defined as immediate restoration of normal sinus rhythm (NSR) after shock delivery.

### LAAO implantation

All patients underwent implantation of either the Watchman FLX or Watchman FLX Pro device (Boston Scientific). Device sizing and deployment were guided by multiplanar TEE or CT-based preprocedural measurements. Technical success was defined as successful device release with adequate compression and no need for surgical bailout. Operators favored performing DCCV prior to device deployment based on the theoretical consideration that once the LAAO device is implanted, minimizing intracardiac and hemodynamic perturbations may reduce the risk of post-deployment device movement or migration. Although this concept remains speculative, performing DCCV before implantation was thought to avoid potential changes in atrial contractility or pressure that could influence device stability immediately after deployment.

### Postprocedural care and discharge management

Patients were monitored overnight unless same-day discharge criteria were met. Postprocedural rhythm was documented before discharge and on postoperative day 1 when available. Antithrombotic therapy at discharge was individualized based on bleeding history, procedural findings, and operator preference, typically consisting of either OAC (eg, warfarin) or dual antiplatelet therapy (DAPT). No patient was discharged without any form of antithrombotic therapy.

### Follow-up protocol

Follow-up imaging with either cardiac CT or TEE was performed at 6 weeks or 3 months, per institutional standards. Imaging assessments included evaluation for DRT, PDL, device compression, and device position. Management changes, including escalation to OAC for DRT, were recorded.

Clinical follow-up was performed at 45 days, 3 months, and 1 year using medical record review. Outcomes included death, cardiovascular death, stroke or systemic embolism, bleeding events, heart failure hospitalization, AF recurrence, repeat cardioversion, new AF ablation, and antithrombotic therapy status.

### Outcomes and definitions

The primary end point was procedural feasibility and safety, defined as (1) successful LAAO device implantation and (2) acute restoration of NSR when intended. Secondary end points included (1) periprocedural complications, (2) DRT or PDL on follow-up imaging, and (3) major adverse events at 45 days, 3 months, and 1 year.

DRT was characterized by established morphological criteria and classified as ‘low-risk’ or ‘high-risk’ morphology.^12^ PDL was categorized as any leak, >3 mm, or >5 mm, consistent with prior LAAO trials. Major bleeding and clinically relevant minor bleeding was defined using VARC-3 criteria.

### Statistical analysis

Continuous variables are presented as mean ± SD or median (IQR), depending on distribution. Categorical variables are expressed as counts. Given the pilot nature and small sample size, no comparative statistics were performed. All analyses were descriptive and performed using Stata 19 (StataCorp).

## Results

**Figure 1:**
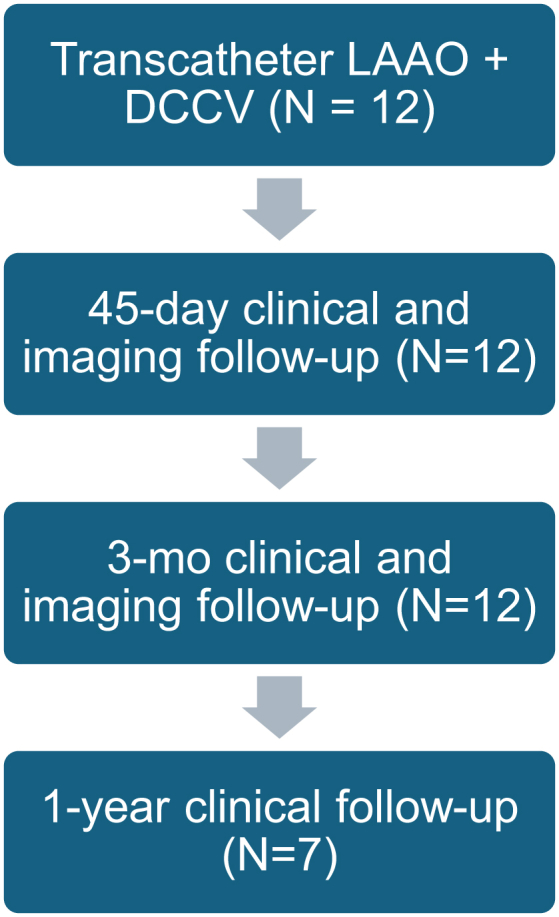
**Participant flow through the combined direct current cardioversion (DCCV) and left atrial appendage occlusion (LAAO) protocol.** All 12 enrolled patients completed 45-day clinical and/or imaging follow-up; 12 patients underwent 3-month imaging follow-up; and 6 patients completed 1-year clinical follow-up.

A total of 12 patients underwent concomitant DCCV + LAAO (Figure 1). Baseline clinical characteristics are summarized in Table 1. Mean age was 75 ± 9 years, 33% were women, and mean body mass index was 27 ± 5 kg/m^2^. A history of congestive heart failure was present in 6 patients (50%), with 2 (17%) having a heart failure hospitalization within the prior year; hypertension and hyperlipidemia were present in 10 (83%) and 9 (75%), respectively, and 4 (33%) had prior stroke or transient ischemic attack. Any prior bleeding was documented in 8 patients (67%), including 1 (8%) with prior major bleeding. Mean CHA_2_DS_2_-VASc and HAS-BLED scores were 3.7 ± 1.1 and 2.1 ± 0.7, respectively.

**Table 1:** Baseline clinical characteristics

Characteristic	N = 12
Age, y	75 ± 9
Female sex	4 (33)
Body mass index, kg/m^2^	27 ± 5
Congestive heart failure	6 (50)
HF hospitalization within prior year	2 (17)
NYHA class III-IV	3 (25)
Hypertension	10 (83)
Diabetes mellitus	1 (8)
Hyperlipidemia	9 (75)
Prior stroke or TIA	4 (33)
CKD stage ≥3	0
Known liver disease	1 (8)
Hemodialysis	0
Vascular disease (including CAD)	5 (42)
Prior AF ablation	2 (17)
Prior cardioversion	3 (25)
Prior pacemaker/ICD/CRT	0
Any prior bleeding	8 (67)
Clinically relevant minor bleeding	7 (58)
Prior major bleeding	1 (8)
CHA_2_DS_2_-VASc score	3.7 ± 1.1
HAS-BLED score	2.1 ± 0.7

Values are mean ± SD or n (%).

AF, atrial fibrillation; CAD, coronary artery disease; CKD, chronic kidney disease; CRT, cardiac resynchronization therapy; HF, heart failure; ICD, implantable cardioverter defibrillator; NYHA, New York Heart Association; TIA, transient ischemic attack.

Baseline arrhythmia, imaging, and structural heart characteristics are detailed in Table 2. Persistent and paroxysmal AF were equally represented (6 [50%] each), with a median AF duration of 2 years (IQR, 1-4). Mean LVEF was preserved (59 ± 5%), with moderate left atrial enlargement (mean left atrial diameter 4.5 ± 0.8 cm; left atrial volume 86 ± 34 mL). Preprocedural OAC was used in 10 patients (83%), and single antiplatelet therapy (SAPT) alone in 2 (17%). Preprocedural imaging was predominantly CT-based (10 [83%]); 2 patients (17%) underwent TEE. No patient had LAA thrombus, while slow flow on CT was observed in 4 (36%). Mean maximal LAA ostial diameter and depth were 26.5 ± 4.3 mm and 27.3 ± 4.5 mm, respectively.

**Table 2:** Baseline arrhythmia and structural heart characteristics

Characteristic	N = 12
AF type	
Persistent	6 (50)
Paroxysmal	6 (50)
AF duration, y	2 (1-4)
Baseline heart rate, bpm	71 ± 12
Preprocedural antiarrhythmic therapy	4 (33)
Preprocedural antithrombotic therapy	
Oral anticoagulation	10 (83)
Single antiplatelet therapy	2 (17)
Left ventricular ejection fraction, %	59 ± 5
LVEDD, mm	48 ± 7
LA diameter, cm	4.5 ± 0.8
LA volume, mL (n = 10)	86 ± 34
RVSP, mm Hg	31 ± 10
Preprocedural imaging	
Computed tomography	10 (83)
Transesophageal echocardiogram	2 (17)
LAA thrombus	0
Slow flow on CT	4 (36)
LAA ostial diameter (max), mm	26.5 ± 4.3
LAA depth (max), mm	27.3 ± 4.5

Continuous variables shown as mean ± SD; categorical variables as n (%).

AF, atrial fibrillation; CT, computed tomography; LA, left atrial; LAA, left atrial appendage; LVEDD, left ventricular end diastolic dimension; RVSP, right ventricular systolic pressure.

Procedural characteristics are shown in Table 3. DCCV was performed before LAAO in 10 patients (83%) and after LAAO in 2 (17%). All patients received a single shock; 11 (92%) were cardioverted with 200 J and 1 (8%) with 150 J. Acute DCCV success, defined as immediate restoration of NSR, was achieved in 11 patients (92%). In the single patient in whom cardioversion was unsuccessful, additional shock attempts were not performed at operator discretion; the rationale for this decision was not clearly documented in the medical record. All patients underwent successful device implantation (technical success 100%), using Watchman FLX in 2 (17%) and Watchman FLX Pro in 10 (83%) patients. Device sizes ranged from 20 to 40 mm; device recapture was required in 3 patients (25%), and >1 device size was attempted in 1 (8%). PDL <3 mm at the index procedure was seen in 2 patients (17%). Mean procedure time from venous puncture to venous closure was 48 ± 15 minutes, with a mean fluoroscopy time of 14 ± 4 minutes, contrast volume of 108 ± 44 mL, and dose–area product of 850 ± 480 Gy·cm^2^. General anesthesia and TEE guidance were used in 11 patients (92% each) and intracardiac echocardiography in 2 (17%); additional intraprocedural antiarrhythmic therapy was given in 3 (25%). At the end of the procedure, 11 patients (92%) were in NSR and 1 (8%) remained in AF; among those with next-day electrocardiograms, 5 of 5 were in NSR. At discharge, 7 patients (58%) were prescribed antiarrhythmic therapy. Furthermore, 10 patients (83%) were discharged with DAPT and 2 (17%) with OAC; no patient was discharged on SAPT alone or without antithrombotic therapy.

**Table 3:** Procedural characteristics

Characteristic	N = 12
DCCV performed before LAAO	10 (83)
Shocks per procedure	1 (all patients)
Maximum DCCV energy	
200 J	11 (92)
150 J	1 (8)
Acute DCCV success	11 (92)
Post-DCCV heart rate, bpm	69 ± 14
Procedure time (VP→VC), min	48 ± 15
Fluoroscopy time, min	14 ± 4
Contrast volume, mL	108 ± 44
Dose–area product, Gy·cm^2^	850 ± 480
General anesthesia	11 (92)
TEE guidance	11 (92)
ICE guidance	2 (17)
Additional antiarrhythmic therapy	3 (25)
Device type	
Watchman FLX	2 (17)
Watchman FLX Pro	10 (83)
Device recapture required	3 (25)
>1 device size attempted	1 (8)
PDL <3 mm at index procedure	2 (17)
Postprocedural NSR	11 (92)
Discharge antithrombotic therapy	
Oral anticoagulation	2 (17)
Dual antiplatelet therapy	10 (83)

Values are mean ± SD or n (%).

DCCV, direct current cardioversion; LAAO, left atrial appendage occlusion; ICE, intracardiac echo; NSR, normal sinus rhythm; PDL, peri-device leak; TEE, transesophageal echo; VC, venous closure; VP, venous puncture.

In-hospital outcomes are summarized in Table 4. Technical LAAO success remained 100%, with acute DCCV success of 92%. Median hospital length of stay was 1 day (IQR, 0-1), with a range of 0 to 4 days, and 5 patients (42%) were discharged the same day.

**Table 4:** In-hospital outcomes

Outcome	N = 12
Technical LAAO success	12 (100)
Acute DCCV success	11 (92)
Length of stay, d	1 (0-1)
Same-day discharge	5 (42)

Values are median (IQR) or n (%).

DCCV, direct current cardioversion; LAAO, left atrial appendage occlusion.

Follow-up imaging findings are shown in Table 5. All 12 patients underwent follow-up CT or TEE at either 6 weeks (3 patients, 25%) or 3 months (9 patients, 75%); CT was used in 11 (92%) and TEE in 1 (8%). A single case of DRT (9%) was identified at 3-month CT imaging, characterized as low-risk morphology and managed with escalation to OAC (the patient was originally discharged on DAPT for 3 months after DCCV/LAAO). PDL >3 mm was present in 2 patients (17%), and no patient had PDL >5 mm. The presence of flow into the appendage (either from device porosity or a PDL <5 mm) was seen in 50% of the overall cohort. After follow-up imaging, 10 patients (84%) were maintained on SAPT, 1 (8%) on DAPT, and 1 (8%) on OAC for management of DRT.

**Table 5:** Follow-up imaging findings

Finding	N = 12
Imaging modality	
Computed tomography	11 (92)
Transesophageal echocardiography	1 (8)
Imaging time point	6 wk: 3; 3 mo: 9
Any DRT	1 (9)
DRT phenotype	Low-risk
OAC escalation for DRT	1 (9)
PDL >3 mm	2 (17)
PDL >5 mm	0
Post-imaging antithrombotic therapy	
SAPT	9 (82)
DAPT	1 (9)
OAC	1 (9)

Values are reported as n (%).

DAPT, dual antiplatelet therapy; DRT, device-related thrombus; OAC, oral anticoagulation; PDL, peri-device leak; SAPT, single antiplatelet therapy.

Clinical outcomes at 45 days, 3 months, and 1 year are presented in Table 6 and summarized in the Central Illustration. At both 45 days and 3 months (N = 12), there were no deaths, strokes, systemic embolic events, hemorrhagic strokes, or major bleeding events. One patient had a heart failure hospitalization by 45 days and no patient by 3 months. AF recurrence occurred in 1 patient at 45 days and in 2 patients at 3 months; 1 patient underwent repeat DCCV by 3 months, and no patient underwent AF ablation. At 1 year (N = 7), there was 1 cardiovascular death (acute coronary syndrome) and 1 heart failure hospitalization, with no stroke, systemic embolism, or major bleeding events observed.

**Table 6:** Clinical outcomes at 45 days, 3 months, and 1 year

Outcome	45 d (n = 12)	3 mo (n = 12)	1 y (n = 7)
Death (any cause)	0	0	1
Cardiovascular death	0	0	1
Stroke/systemic embolism	0	0	0
Major bleeding	0	0	0
HF hospitalization	1	0	1
AF recurrence	1	2	1
Repeat DCCV	0	1	0
New AF ablation	0	0	0

Values are reported as n.

AF, atrial fibrillation; DCCV, direct current cardioversion; HF, heart failure.

**Central Illustration:**
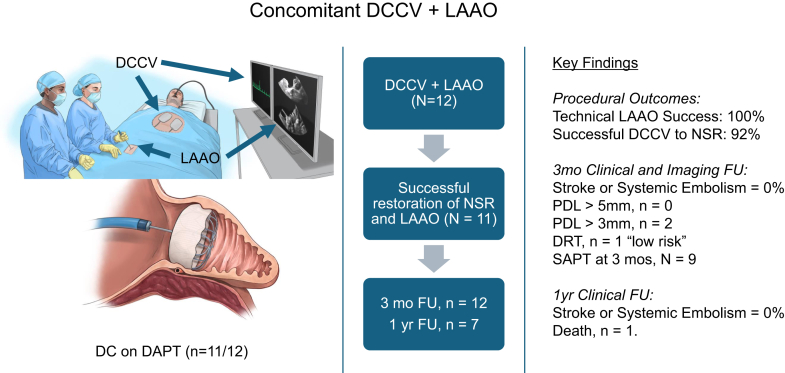
**The combined procedural approach, demonstrating immediate DCCV followed by percutaneous LAAO, postprocedural restoration of sinus rhythm, and structured imaging and clinicalfollow-up.** Key results include 100% technical LAAO success, 92% acute cardioversion success, low-risk DRT in 1 patient, no PDL >5 mm, and no stroke or systemic embolism through 3 months, with 1 cardiovascular death by 1 year. DAPT, dual antiplatelet therapy; DC, direct current; DCCV, direct current cardioversion; DRT, device-related thrombus; FU, follow-up; LAAO, left atrial appendage occlusion; NSR, normal sinus rhythm; PDL, peri-device leak; SAPT, single antiplatelet therapy.

## Discussion

In this pilot series, we describe a combined strategy of DCCV immediately followed by LAAO in patients with AF and relative or absolute contraindications to systemic OAC. The key observation is that this "one-stop" approach was feasible, procedurally successful, and not associated with excess thromboembolic or bleeding events during early and intermediate follow-up (Central Illustration). Although limited by small sample size and retrospective design, these findings support the concept that rhythm restoration and stroke prophylaxis can be safely achieved in a single session in carefully selected patients.

Several prior investigations have examined the safety of DCCV in patients after LAAO, typically in cohorts in whom the device was already implanted and cardioversion was later required for recurrent AF. Sharma and colleagues^13^ reported that DCCV in patients with Watchman devices was feasible and safe, with no increase in stroke when TEE excluded DRT and major PDL, even in the absence of systemic OAC. More recent multicenter and registry data have similarly shown that elective cardioversion in patients with prior LAAO is associated with very low thromboembolic rates, albeit with heterogeneous imaging and anticoagulation strategies.^9^^,^^14^^,^^15^ Our series differs in that cardioversion and device implantation were intentionally combined in the same setting, with LAAO used as a substitute for the conventional 3- to 4-week period of mandatory OAC after cardioversion. Although this strategy does not eliminate the theoretical risk of non-LAA thrombus formation, no thromboembolic events were observed in our cohort. Additionally, CT analysis at 3 months (92% of patients) did not demonstrate non-LAA thrombus.

The concept of consolidating AF rhythm control and LAAO is not entirely new; multiple groups have reported on one-stop AF ablation plus LAAO procedures. Concomitant catheter ablation and LAAO in a single session is technically feasible, with acceptable complication rates and favorable medium-term outcomes in high-risk populations,^16^, ^17^, ^18^ Our study extends this combined-procedure paradigm specifically to DCCV + LAAO in patients who are poor candidates for post-cardioversion anticoagulation in the contemporary Watchman FLX/FLX Pro era. The absence of stroke, systemic embolism, or major bleeding in this cohort suggests that combining the 2 procedures is not associated with an obvious safety signal, although definitive conclusions will require larger prospective studies.

An important concern historically has been electromechanical dissociation of the left atrium and LAA after cardioversion, with transient atrial stunning predisposing to thrombus formation even when sinus rhythm has been restored. This pathophysiologic rationale underpins guideline recommendations for at least 3 to 4 weeks of systemic anticoagulation following cardioversion in most patients with AF of ≥48 hours duration.^2^^,^^19^ In our pilot experience, however, LAAO was performed in the same procedural episode as DCCV, and patients were managed predominantly with DAPT rather than OAC thereafter. Despite this, no thromboembolic events were observed during the 45-day and 3-month follow-ups, and the single DRT detected on CT was designated as ”low-risk“ based on accepted morphologic criteria and treated with escalation to direct OAC.^20^ Given that DRT rates of 3% to 4% are reported even in standard LAAO populations treated according to guideline-directed antithrombotic protocols,^21^^,^^22^ the single low-risk DRT event in our series likely reflects the baseline procedural and patient ‘protoplasm’ risk rather than a specific hazard of the combined approach.

Our LAAO outcomes otherwise mirror contemporary experience. Technical success was 100%, periprocedural complications were absent, and most patients were discharged on DAPT, in line with modern registries of patients with high bleeding risk treated with Watchman FLX.^6^^,^^7^ Of particular note, follow-up imaging was almost exclusively CT-based. Prior work has shown that CT is more sensitive than TEE for detecting small PDLs and for characterizing device apposition and compression.^23^^,^^24^ The low prevalence of leaks >3 mm and absence of leaks >5 mm is very similar to CT-based series and likely reflects enhanced detection rather than clinically meaningful device failure. Importantly, we did not observe a corresponding increase in stroke or systemic embolism, supporting the growing notion that small CT-detected PDLs may not uniformly translate into adverse events, especially when patients remain on at least antiplatelet therapy.

Another distinguishing feature of this cohort is the systematic CT follow-up—rather than selective TEE in symptomatic or high-suspicion cases—which provides a more granular view of device healing and thrombotic risk after combined DCCV + LAAO. Recent reports have highlighted wide variation in imaging and anticoagulation practices when cardioversion is performed in patients with prior LAAO, with pre-DCCV imaging identifying left atrial thrombus or DRT in ∼6% of patients in some series.^9^^,^^13^, ^14^, ^15^ In contrast, our approach applied the ”imaging rigor“ of contemporary LAAO programs to the combined-procedure setting and still did not reveal a signal for excess thrombus or stroke.

Taken together, these data suggest that combining DCCV and LAAO in a single session is associated with procedural performance and feasibility comparable to standalone LAAO, while potentially obviating the need for a prolonged course of systemic anticoagulation after cardioversion in patients at high bleeding risk. However, because this is a small, nonrandomized, retrospective series, the findings should be interpreted strictly as associative, not causal. The present study is best viewed as hypothesis-generating and supports the need for larger, multicenter prospective studies comparing combined versus staged DCCV and LAAO strategies, ideally with standardized imaging and antithrombotic protocols and longer-term follow-up.

### Limitations

This study has several important limitations. First, it is a retrospective analysis from a single tertiary center, limiting generalizability and precluding determination of causality. Second, the sample size is small, and only a subset of patients (50%) had completed 1-year follow-up at the time of analysis, with findings remaining exploratory with cautious interpretation. Third, although CT imaging provides superior spatial resolution, its high sensitivity for detecting small PDLs may influence interpretation of PDL incidence relative to prior TEE-based studies. Fourth, the choice of antithrombotic regimen, timing of cardioversion, and use of antiarrhythmic therapy were operator-dependent and not standardized. Finally, the study lacked a comparison group of patients undergoing staged DCCV followed by later LAAO, limiting our ability to evaluate relative benefits or risks.

### Conclusion

In this pilot experience, a combined procedural strategy of DCCV immediately followed by LAAO was feasible, well tolerated, and not associated with increased thromboembolic or bleeding complications. Procedural outcomes, discharge antithrombotic regimens, and follow-up imaging findings—including PDL and DRT rates—were comparable to those reported in contemporary LAAO practice. These results suggest that concomitant DCCV and LAAO may represent a reasonable alternative for patients in whom systemic anticoagulation after cardioversion is contraindicated. Larger, prospective studies are needed to validate these findings and to better define the role of combined rhythm restoration and appendage closure in clinical practice.

## CRediT authorship contribution statement

**Lawrence Chen:** Data curation, Writing – original draft, Writing – review & editing, Conceptualization, Investigation, Methodology, Validation. **Tai Pham:** Data curation, Supervision, Writing – review & editing. **Edris Aman:** Data curation, Supervision, Writing – review & editing. **Anthony Khong:** Data curation, Writing – review & editing. **Irsa Munir:** Data curation, Supervision, Writing – review & editing. **Tiffany Dong:** Data curation, Supervision, Writing – review & editing. **Muhammad B. Munir:** Data curation, Writing – review & editing. **Thomas W.R. Smith:** Supervision, Writing – review & editing. **Jason H. Rogers:** Writing – review & editing. **Gagan Singh:** Conceptualization, Formal analysis, Investigation, Methodology, Supervision, Validation, Writing – original draft, Writing – review & editing.
